# In vitro anti-tumour activity of α-galactosylceramide-stimulated human invariant Vα24+NKT cells against melanoma

**DOI:** 10.1054/bjoc.2001.1973

**Published:** 2001-09

**Authors:** A Kikuchi, M Nieda, C Schmidt, Y Koezuka, S Ishihara, Y Ishikawa, K Tadokoro, S Durrant, A Boyd, T Juji, A Nicol

**Affiliations:** Department of Research, The Japanese Red Cross Central Blood Center, 4-1-31 Hiroo, Shibuya-ku, Tokyo, 150-0012, Japan; Leukaemia Foundation of Queensland Laboratory, Queensland Institute of Medical Research, Herston Road, Herston, Brisbane, 4029; Pharmaceutical Research Laboratory, Kirin Brewery, 3 Miyahara Takasaki, Gunma, 370-1295, Japan; Department of Surgery, Division of Surgical Oncology, Tokyo University School of Medicine, 7-3-1 Hongo, Bunkyo-ku, Tokyo, 113-8655, Japan; Department of Medicine, The University of Queensland, Royal Brisbane Hospital, C floor Clinical Sciences Building, Herston Rd, Herston Brisbane, 4029, Australia

**Keywords:** melanoma, anti-tumour activity, Vα24+NKT-cells, α-GalCer, IFN-γ

## Abstract

α-galactosylceramide (KRN 7000, α-GalCer) has shown potent in vivo anti-tumour activity in mice, including against melanoma and the highly specific effect of inducing proliferation and activation of human Vα24+NKT-cells. We hypothesized that human Vα24+NKT-cells activated by α-GalCer might exhibit anti-tumour activity against human melanoma. To investigate this, Vα24+NKT-cells were generated from the peripheral blood of patients with melanoma after stimulation with α-GalCer pulsed monocyte-derived dendritic cells (Mo-DCs). Vα24+NKT-cells did not exhibit cytolytic activity against the primary autologous or allogeneic melanoma cell lines tested. However, proliferation of the melanoma cell lines was markedly suppressed by co-culture with activated Vα24+NKT-cells (mean ± SD inhibition of proliferation 63.9 ± 1.3%). Culture supernatants of activated Vα24+NKT-cell cultures stimulated with α-GalCer pulsed Mo-DCs exhibited similar antiproliferative activities against melanoma cells, indicating that the majority of the inhibitory effects were due to soluble mediators rather than direct cell-to-cell interactions. This effect was predominantly due to release of IFN-γ, and to a lesser extent IL-12. Other cytokines, including IL-4 and IL-10, were released but these cytokines had less antiproliferative effects. These in vitro results show that Vα24+NKT-cells stimulated by α-GalCer-pulsed Mo-DCs have anti-tumour activities against human melanoma through antiproliferative effects exerted by soluble mediators rather than cytolytic effects as observed against some other tumours. Induction of local cytokine release by activated Vα24+NKT-cells may contribute to clinical anti-tumour effects of α-GalCer. © 2001 Cancer Research Campaign http://www.bjcancer.com


*British Journal of Cancer* (2001) **85**(5), 741–746
© 2001 Cancer Research Campaign
doi: 10.1054/ bjoc.2001.1973, available online at http://www.idealibrary.com on IDEAL

http://www.bjcancer.com

### In vitro anti-tumour activity of α-galactosylceramide-stimulated human invariant Vα24+NKT cells against melanoma

**A Kikuchi[1,2], M Nieda[1], C Schmidt[2], Y Koezuka[3], S Ishihara[4], Y Ishikawa[1], K Tadokoro[1], S Durrant[5], A Boyd[2], T Juji[1] and A Nicol[2,5]**

[1]Department of Research, The Japanese Red Cross Central Blood Center, 4-1-31, Hiroo, Shibuya-ku, Tokyo 150-0012, Japan; [2]Leukaemia Foundation of Queensland Laboratory, Queensland Institute of Medical Research, Herston Road, Herston, Brisbane 4029; [3]Pharmaceutical Research Laboratory, Kirin Brewery, 3 Miyahara, Takasaki, Gunma, 370-1295, Japan; [4]Department of Surgery, Division of Surgical Oncology, Tokyo University School of Medicine, 7-3-1 Hongo, Bunkyo-ku, Tokyo 113-8655, Japan; [5]Department of Medicine, The University of Queensland, C floor Clinical Sciences Building, Royal Brisbane Hospital, Herston Rd, Herston Brisbane 4029, Australia

**Summary** α-galactosylceramide (KRN 7000, α-GalCer) has shown potent in vivo anti-tumour activity in mice, including against melanoma and the highly specific effect of inducing proliferation and activation of human Vα24+NKT-cells. We hypothesized that human Vα24+NKT-cells activated by α-GalCer might exhibit anti-tumour activity against human melanoma. To investigate this, Vα24+NKT-cells were generated from the peripheral blood of patients with melanoma after stimulation with α-GalCer pulsed monocyte-derived dendritic cells (Mo-DCs). Vα24+NKT-cells did not exhibit cytolytic activity against the primary autologous or allogeneic melanoma cell lines tested. However, proliferation of the melanoma cell lines was markedly suppressed by co-culture with activated Vα24+NKT-cells (mean ± SD inhibition of proliferation 63.9 ± 1.3%). Culture supernatants of activated Vα24+NKT-cell cultures stimulated with α-GalCer pulsed Mo-DCs exhibited similar antiproliferative activities against melanoma cells, indicating that the majority of the inhibitory effects were due to soluble mediators rather than direct cell-to-cell interactions. This effect was predominantly due to release of IFN-γ, and to a lesser extent IL-12. Other cytokines, including IL-4 and IL-10, were released but these cytokines had less antiproliferative effects. These in vitro results show that Vα24+NKT-cells stimulated by α-GalCer-pulsed Mo-DCs have anti-tumour activities against human melanoma through antiproliferative effects exerted by soluble mediators rather than cytolytic effects as observed against some other tumours. Induction of local cytokine release by activated Vα24+NKT-cells may contribute to clinical anti-tumour effects of α-GalCer. © 2001 Cancer Research Campaign http://www.bjcancer.com

**Keywords**: melanoma, anti-tumour activity, Vα24+NKT-cells, α-GalCer, IFN-γ

α-GalCer has been shown to be a specific and powerful activator of murine Vα14+NKT-cells. α-GalCer and Vα14+NKT-cells have been shown to have potent anti-tumour activities against a range of malignancies, including melanoma, both in vitro and in vivo in mice (Burdin et al, 1999; Kawano et al, 1998; Kobayashi et al, 1995). α-GalCer also specifically activates and induces proliferation of human invariant Vα24+NKT-cells, a subpopulation of NK-cell receptor (NKR-P1A)-expressing T-cells with an invariant T-cell receptor (Vα24JαQ), which are the human counterpart of murine Vα14+NKT-cells (Dellabona et al, 1994). Activation of invariant Vα24+NKT-cells by α-GalCer is CD1d dependent and TCR mediated, but is MHC independent (Couedel et al, 1998; Kawano et al, 1997; Nieda et al, 1999). The anti-tumour activity and potential for clinical use of α-GalCer in humans are under investigation. We are examining whether α-GalCer and human invariant Vα24+NKT-cells have anti-tumour activities in humans and also the mechanisms of any apparent anti-tumour activity of these cells. On the basis of in vivo observations in murine systems, we hypothesized that α-GalCer might exhibit anti-tumour activity against human melanoma and that human Vα24+NKT-cells activated by α-GalCer-pulsed Mo-DCs might mediate at least some of these activities (Cui et al, 1997).

Human invariant Vα24+NKT-cells activated by α-GalCer have been shown to have cytotoxic activity in a number of studies (Couedel et al, 1998; Kawano et al, 1999). They exhibit TCR-mediated cytotoxic activity against CD1d-expressing cells. This might represent a form of autoreactivity or occur through recognition of an unknown endogenous ligand. In either case, it is unlikely that broad-spectrum anti-tumour cytolytic activity would be exerted directly through TCR-mediated recognition of CD1d, as CD1d is expressed only on a limited number of cell lineages (Calabi F and Bradbury 1991). The anti-tumour activity of Vα24+NKT-cells is thus likely to be exerted through alternative cytotoxic mechanisms. In support of this, we have previously shown that invariant Vα24+NKT-cells exhibit cytolytic anti-tumour activities against some tumour cell lines that do not express CD1d through killing mechanisms that are distinct from both those of NK-cells and T-cells (Nicol et al, 1999). Also, the presence of NKR-P1A receptors, which may be linked to the cytolytic activity, argues for an important cytotoxic function of human invariant Vα24+NKT-cells other than that exerted through TCR-mediated recognition of CD1d (Azzoni et al, 1998; Bezouska et al, 1994). Alternatively, the anti-tumour effects could be exerted through inhibition of proliferation, either by direct

*Received 24 October 2000*
*Revised 14 May 2001*
*Accepted 16 May 2001*

*Correspondence to:* A Nicol cell–cell contact or via the release of soluble factors, including inhibitory cytokines. To investigate these possibilities, we examined the cytolytic and anti-proliferative activities of human invariant Vα24+NKT-cells against human primary melanoma cell lines.

### MATERIALS AND METHODS

### Human invariant Vα24+NKT-cells

Invariant Vα24+NKT-cells were established as follows. Fresh mononuclear cells from patients with melanoma (I1 and J1) were incubated for 2 h to separate the adherent and non-adherent cells. The adherent cells (> 90% monocytes) were then cultured with 400 U/ml rhIL-4 (Schering Plough) and 800 U/ml rhGM-CSF (Schering Plough) for 5–7 days to produce Mo-DCs. The non-adherent cells were cultured with irradiated (30 Gy) Mo-DCs, pulsed with 100 ng/ml α-GalCer (Kirin Corp, Gunma, Japan), and maintained by re-stimulation every 7–10 d with α-GalCer-pulsed Mo-DCs. After the second stimulation, invariant Vα24+NKT cells were separated by positive (Vα24TCR) magnetic bead sorting (miniMACS, Miltenyi Biotec, Gladbach, Germany). After further expansion induced by repeated stimulation with α-GalCer-pulsed Mo-DC, Vα24+CD161+CD4+ cells were obtained by positive cell selection for CD161 by immunomagnetic separation (miniMACS, Miltenyi Biotec) and flow cytometry (FACS Vantage, Becton Dickinson, CA, USA).

### Phenotypic analysis

The cell surface phenotype of the cells expanded in response to α-GalCer was determined by single-and 2-colour flow cytometry. The following monoclonal antibodies (mAbs) were obtained from Immunotech: FITC-anti-CD3 (UCHT1), anti-CD3 (X35), FITC-anti-CD4 (13B8.2), PE-anti-CD8 (B9.11), FITC-anti-Vα 24 (C15), anti-Vβ11 (c21)PE-anti-P58.1 (EB6), FITC-anti-p58.2 (GL183), anti-CD16 (3G8), anti-CD56 (N901), PE-anti-CD94 (HP-3B1). PE-anti-P70 (NKB1; DX9) and anti-NKR-P1A (DX12) were obtained from Becton Dickinson.

### Target melanoma cell lines

Primary melanoma cell lines (MO3, MO8 and MO9) were established from metastatic deposits in patients (*n* = 3) with stage 4 melanoma. Phenotypic analysis using antihuman CD1d monoclonal antibodies (CD1d51.1) confirmed that the cell lines were CD1d negative (data not shown).

### Proliferation assays

The effect on proliferation of the melanoma cell line (M09) of its co-culture with Vα24+NKT-cells, α-GalCer-pulsed Mo-DCs, culture supernatants (see later) or cytokines, was assessed using thymidine uptake assays as follows. Irradiated (30 Gy) Vα24+NKT-cells ($1 \times 10^4$ cells/well) and α-GalCer-pulsed Mo-DCs ($5 \times 10^3$ cells/well) were cultured with the primary melanoma cell line, M09 ($5 \times 10^3$ or $2.5 \times 10^3$ cells) for 96 h. During the final 18 h of incubation, 1uCi[$^3$H] thymidine ($^3$H-TdR) was added to each well. The incorporation of $^3$H-TdR was determined by liquid scintillation counting. The results are expressed as the mean ± SD counts min for 3 cultures. Melanoma cells ($1 \times 10^4$) were cultured with and without 50 ul of the supernatant in a total volume of 200 ul in 96-well flat-bottom plates for 72 h. Thymidine uptake was assessed as mentioned earlier.

### Analysis of culture supernatants

To obtain supernatants, Vα24+NKT cells ($2 \times 10^5$/ml) cultured with and without α-GalCer-pulsed Mo-DCs ($1 \times 10^5$/ml) were suspended in 1 ml AIM-V medium containing 10% AB serum and cultured in 24-well plates. Supernatants were collected after 2, 3 and 4 d. The concentrations of IFNγ, IL-4, IL-10 and IL-12 in the supernatants were measured by enzyme-linked immunosorbent assay (ELISA) (Immunotech, Marseille, France). The effects of the supernatants on melanoma cell proliferation were assessed as described earlier. To examine the role of the individual cytokines in the supernatants on melanoma cell proliferation, the supernatants or medium were pre-treated with mAb (10 ug/ml) for 30 min before being added to the 3 melanoma cell lines ($1 \times 10^4$), which were then cultured for 96 h. Thymidine uptake was assessed as mentioned earlier.

### RESULTS

### Phenotype of invariant Vα24+NKT-cells established from patients with melanoma

Cells from patients with melanoma (*n* = 2, J1 and I1) responding to α-GalCer-pulsed Mo-DCs (CD1d positive, lineage marker negative) and selected for expression of Vα24+ TCR were predominantly CD3+, CD4+, CD8–, CD161+ (NKR-P1A), Vβ11+ and negative for Ig-type NK receptors such as p58.1, p58.2 and p70, the lectin-type NK receptor CD94 and other markers for NK cells including CD16 and CD56 (Table 1). This phenotype, observed for both the cases of metastatic melanoma examined, was the same as that of cells similarly derived from normal donors (Takahashi et al, 2000). The proliferative response of invariant Vα24+NKT-cells to autologous Mo-DCs was enhanced by the addition of α-GalCer (data not shown). Vα24+NKT-cells did not exhibit any cytolytic activity against allogeneic and autologous primary melanoma cell lines (data not shown).

**Table 1** Phenotypic analysis of purified Vα24+ CD4+ NKT-cells derived from peripheral blood of patients with matastatic melanoma

| T-cell receptors | |
|---|---|
| Vα24 | + |
| Vβ11 | + |
| NK receptors | |
| CD161 | – |
| p70 | – |
| CD94 | – |
| p58.1 | – |
| p58.2 | – |
| T-cell markers | |
| CD3 | + |
| CD4 | + |
| CD8 | – |
| NK markers | |
| CD16 | – |
| CD56 | – |

*© 2001 Cancer Research Campaign*

BJOC 01-1973 741-746 20/8/01 3:31 pm Page 743

### Cytotoxic activity of invariant Vα24+NKT-cells established from patients with melanoma

Invariant Vα24+NKT-cells did not exhibit any cytolytic activity against autologous or 3 allogeneic primary melanoma cell lines, or against the NK target K562. All assays were undertaken at E/T ratios of 10:1, 20:1 and 50:1, using a conventional 4 h $^{51}$Cr release assay (data not shown). As a positive control for the $^{51}$Cr release assays, we confirmed that the invariant Vα24+NKT-cell lines evaluated had cytolytic activity against the U937 cell line, previously shown to be highly sensitive to invariant Vα24+NKT-cell killing (data not shown).

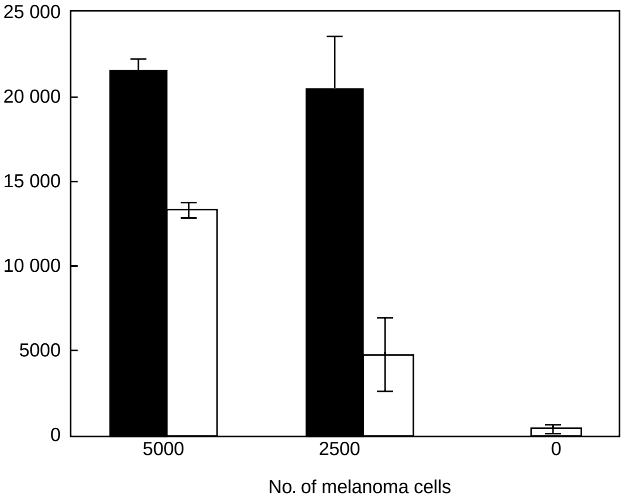

**Figure 1** Inhibition of the proliferation of a melanoma cell line (M09) by activated Vα24+NKT-cells. Melanoma cells cultured alone are shown as black bars and melanoma cells cultured in the presence of activated Vα24+NKT-cells are shown as white bars. Results are shown as the mean ± SD for 3 cultures

### Antiproliferative effects of invariant Vα24+NKT-cells on melanoma cells

Co-culture of invariant Vα24+NKT-cells (J1) and α-GalCer-pulsed Mo-DCs with melanoma cells resulted in marked inhibition of the proliferation of melanoma cells (M09) (Figure 1). We assessed whether this inhibition required direct cell–cell contact between invariant Vα24+NKT-cells and melanoma cells or resulted from soluble factors released by the invariant Vα24+NKT-cells. The effect of the culture supernatants of invariant Vα24+NKT-cells on melanoma cell proliferation (MO3, MO8 and MO9) was assessed. Culture supernatants of invariant Vα24+NKT-cells stimulated with α-GalCer-pulsed Mo-DCs produced approximately 70% inhibition of melanoma cell proliferation. This was similar to the results of the direct co-culture of activated Vα24+NKT-cells with target melanoma cells (Figure 1). In contrast, supernatants of invariant Vα24+NKT-cells cultured with α-GalCer but not Mo-DCs (V2, V3 and V4) and of Mo-DCs cultured in the presence of α-GalCer but not Vα24+NKT-cells (M3) did not inhibit melanoma cell proliferation (Figure 2). These results confirm that the inhibitory effects were due to soluble factors released during the interaction between invariant Vα24+NKT-cells and α-GalCer-pulsed Mo-DCs. These soluble factors were not released by α-GalCer-pulsed Mo-DCs alone and the effects were also not observed with α-GalCer alone.

### Release of inhibitory cytokines

To determine whether this antiproliferative activity resulted from the release of cytokines known to have the potential to inhibit tumour cell proliferation, we first assessed the levels of IFN-γ, IL-4, IL-10 and IL-12 (Figure 3A–D) in the supernatants of invariant Vα24+NKT-cell cultures. Supernatants of Vα24+NKT-cells co-cultured with α-GalCer-pulsed Mo-DCs were compared with those of Vα24+NKT-cells cultured alone. Higher levels of IFN-γ, IL-4 and IL-10 were detected in the supernatants of the Vα24+NKT-cells co-cultured with α-GalCer-pulsed Mo-DCs than in those of Vα24+NKT-cells cultured alone. IL-12 was detected

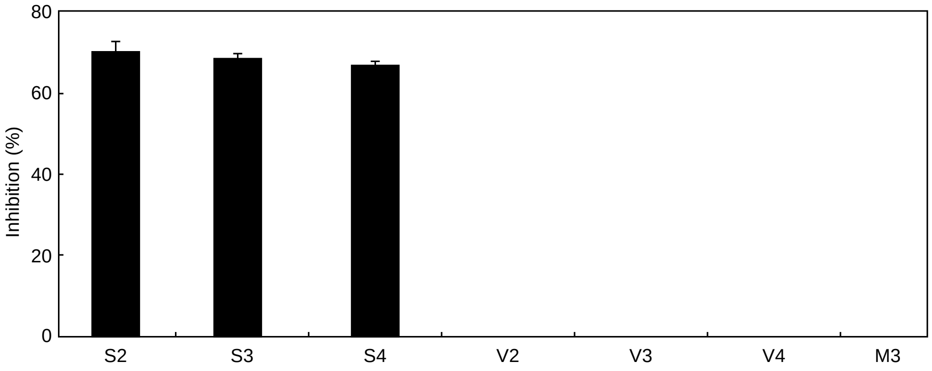

**Figure 2** Inhibition of the proliferation of melanoma cells by various supernatants. S2, S3 and S4 are supernatants from Vα24+NKT-cells cultured with α-GalCer-pulsed autologous Mo-DCs for 2, 3 and 4 days, respectively. V2, V3, V4 are supernatants harvested from 2-, 3-, and 4-day cultures of Vα24+NKT-cells without α-GalCer-pulsed Mo-DCs. M3 is supernatant harvested after 3 days of α-GalCer-pulsed Mo-DC culture. Inhibition of proliferation is given as a percentage of the proliferation with no culture supernatant calculated as follows: (cpm proliferation in medium alone – cpm experimental proliferation)/cpm proliferation in medium alone × 100. Results are shown as the mean ± SD for 3 cultures

© 2001 Cancer Research Campaign

BJOC 01-1973 741-746 20/8/01 3:31 pm Page 744

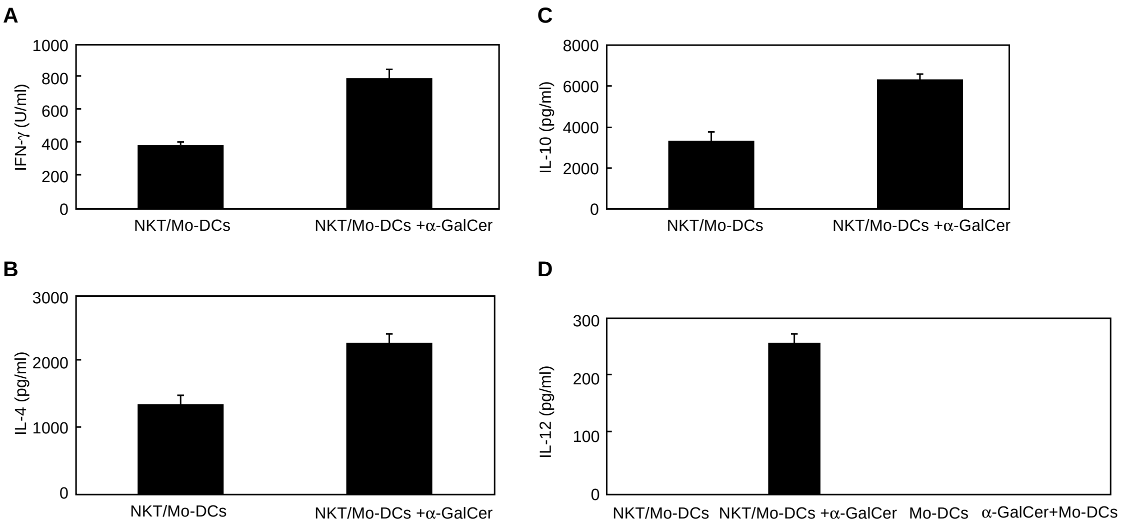

**Figure 3** Cytokine production, assessed by the level of cytokine in culture supernatants, during co-culture of Vα24+NKT-cells with and without α-GalCer-pulsed Mo-DCs

**Table 2** Effects of recombinant cytokines on melanoma cell growth

| | percentage of proliferation | | | | | | | |
|---|---|---|---|---|---|---|---|---|
| | **cytokines** | | | | | | | |
| | **IFNY** | | **IL-12** | | **IL-4** | | **IL-10** | |
| | **60 U/ml** | **600 U/ml** | **150 pg/ml** | **1500 pg/ml** | **2 ng/ml** | **20 ng/ml** | **10 ng/ml** | **100 ng/ml** |
| Allogenic M03 | 55.1±14.0 | 39.5±3.0 | 112.9±29.2 | 63.4±1.6 | 90.5±20.9 | 155.4±23.9 | 85.3±35.3 | 99.2±7.6 |
| Autologous M08 | 52.3±11.2 | 39.7±3.8 | 82.9±14.4 | 75.0±6.3 | 104.7±31.2 | 113.8±27.5 | 92.9±0.5 | 83.7±0.9 |
| Allogenic M09 | 45.6±11.2 | 47.6±3.2 | 90.4±8.8 | 74.3±14.3 | 150.3±5.7 | 126.0±12.4 | 93.8±27.5 | 86.8±0.6 |

$1 \times 10^4$ melanoma cells were cultured in the absence or presence of IFN-γ (60 U/ml, 600 U/ml), IL-10 (10 ng/ml,100 ng/ml) and IL-12 (150 pg/ml, 1500 pg/ml) for 72h. [$^3$H] thymidine was added to each well for the final 18 h of incubation. The percentage proliferation was calculated as follows: cpm experimental proliferation/cpm proliferation/cpm proliferation in medium alone × 100. Results are shown as the mean ± SD for 3 cultures.

**Table 3** Blocking effects of MAbs on culture supernatant inhibition of melanoma cell growth

| | | Blocking antibody | | | |
|---|---|---|---|---|---|
| **Melanoma cells** | **None** | **α-IFNγmAB (IgG2)** | **α-IL-12mAb (IgG1)** | **Isotype control** | |
| | | | | **α-CD3mAB(IgG2)** | **Mouse IgG1** |
| M03 | 100 | 0.8 ± 14.6 | 93.16 ± 7.3 | 91.4 ± 18.6 | 120.0 ± 2.2 |
| M08 | 100 | 0.8 ± 18.0 | 103.2 ± 2.3 | 100.7 ± 4.7 | 107.9 ± 6.8 |
| M09 | 100 | 48.7 ± 2.5 | 110.7 ± 4.7 | 105.1 ± 10.7 | 100.9 ± 7.6 |

The results are shown as percentage suppression of proliferation. This was calculated as follows: (cpm proliferation in medium alone – cpm in the presence of the supernatant treated with mAb)/(cpm proliferation in medium alone – cpm in the presence of the supernatant) × 100. Results are shown as the mean ± SD for 3 cultures.

only in the supernatants of Vα24+NKT-cells cultured with α-GalCer-pulsed Mo-DCs but not in those of Vα24+NKT-cells cultured with Mo-DCs or those of α-GalCer-pulsed Mo-DCs and Mo-DCs (Figure 3D).

### Anti-tumour effects of cytokines released in Vα24+NKT-cell cultures

To determine whether the cytokines released by activated Vα24+NKT-cells indeed contribute to their antiproliferative effects, we examined the antiproliferative effects of recombinant

 *© 2001 Cancer Research Campaign*

cytokines (Table 2). IFN-γ was the most potent individual cytokine that could inhibit the proliferation of all the 3 melanoma cell lines tested. IL-12 also exhibited weak antiproliferative activity against melanoma cells. IL-4 and IL-10 had minimal or no effect. Inhibition by the combination of cytokines approached that by the supernatants of activated Vα24+NKT-cells (data not shown). The importance of IFN-γ in the antiproliferative effect exerted by the supernatants of activated Vα24+NKT-cells was assessed by attempting to block the effect with anti-IFN-γ monoclonal antibodies. This resulted in complete blocking of the inhibitory effects of the culture supernatants Vα24+NKT-cells on proliferation of melanoma cells in 2 cases (M03 and M08) and a moderate reduction in the third (M09) (Table 3). As the interactions between α-GalCer-pulsed Mo-DCs and Vα24+NKT-cells were found to be necessary for their exertion of inhibitory effects against melanoma cells and for the release of inhibitory cytokines by the Vα24+NKT-cells, we cultured α-GalCer-pulsed Mo-DCs and Vα24+NKT-cells separately in transwells to determine whether direct cell-to-cell contact was required for these effects. Inhibition of melanoma cell (M09) proliferation by Vα24+NKT-cells and the release of high levels of IFN-γ and IL-10 were only detected when the Vα24+NKT-cells were co-cultured in direct contact with α-GalCer-pulsed Mo-DCs and not when the cells were separated by the porous membrane of the transwell (data not shown).

### DISCUSSION

The results presented here are the first to show that human invariant Vα24+NKT-cells have anti-tumour effects against human melanoma and that activation by α-GalCer-pulsed-dendritic cells is essential for this effect. Our results, using primary melanoma cell lines established from a series of patients with metastatic melanoma, confirmed the potentially important anti-tumour effect of α-GalCer-activated invariant Vα24+NKT-cells. However, contrary to our expectations, the predominant anti-tumour effect on melanoma cells was inhibition of proliferation rather than direct cytotoxic killing. Activation of Vα24+NKT-cells involving direct contact between Vα24+NKT-cells and α-GalCer-pulsed dendritic cells was found to be essential for the induction of cytokine release and antiproliferative anti-tumour activities of the invariant Vα24+NKT-cells or their culture supernatants. Invariant Vα24+NKT-cells are not activated by α-GalCer alone and α-GalCer alone does not appear to have any direct anti-tumour effects under the conditions examined. Supernatants of invariant Vα24+NKT-cells cultured with α-GalCer but not Mo-DCs, and of Mo-DCs cultured in the presence of α-GalCer but not Vα24+NKT-cells, did not inhibit melanoma cell proliferation. The requirement of α-GalCer-pulsed dendritic cells for activation of the inhibitory functions of CD4+ Vα24+NKT-cells strongly suggests that the activation of CD4+ Vα24+NKT-cells occurs via α-GalCer presented by the CD1d expressed on dendritic cells to the Vα24+TCR, as we have previously reported for CD4–CD8- Vα24+NKT-cells. A similar pathway of Vα24+NKT-cell activation is also presumed to occur in vivo, although the natural ligand remains to be identified.

Activated Vα24+NKT-cells do not require direct contact with the target cells to exert their antiproliferative effects, which appear to be mediated through the release of inhibitory cytokines, in particular IFN-γ. Blocking studies confirmed that, among all the factors investigated, IFN-γ contributes the most to the antiproliferative effects of activated invariant Vα24+NKT-cells. IL-12 is also released but appears to contribute less to the less antiproliferative effects under these conditions than IFN-γ. Our results were consistent with previous reports that revealed that IFN-γ is a potent immunomodulator, and exhibits anti-tumour activity against melanoma both in vitro and in murine models in vivo (Fujimoto et al, 1996; Gillis and Williams, 1998). There are many reports that suggest that IL-12 exerts anti-tumour effects (Cui et al, 1997; Kitamura et al, 1999; Yue et al, 1999), however, the underlying mechanisms are still not clear. In our study, IL-12 did not strongly inhibit melanoma cell growth. It has also been reported that IL-10 produced by tumour cells inhibits the synthesis macrophage-derived angiogenic factors, and hence tumour growth and metastasis. In the clinical setting, in patients with melanoma and possibly other malignancies, therapeutic administration of α-GalCer or α-GalCer-pulsed dendritic cells may induce proliferation and activation of Vα24+NKT-cells, resulting in anti-tumour effects analogous to those observed in vitro. Whether there exist anti-tumour effects in addition to those exerted by the release of inhibitory cytokines remains to be confirmed, however activated Vα24+NKT-cells may still have a unique role in anti-tumour immune therapy if they can release cytokines locally in the region of tumour cells, allowing far greater concentrations of IFN-γ, IL-12 or other soluble mediators with anti-tumour activity to accumulate than can be achieved by systemic administration of these agents.

In summary, our results indicate that human invariant Vα24+NKT-cells have anti-tumour effects against human melanoma cells in vitro that could translate into a clinical therapeutic effect. These anti-tumour effects are antiproliferative, rather than cytolytic and result from the release of inhibitory factors during interactions requiring direct contact between the Vα24+NKT-cells and α-GalCer-pulsed dendritic cells. Based on these results we propose that KRN7000 (α-GalCer) may have potential therapeutic value against human melanoma with their effects mediated via invariant Vα24+NKT-cells.

### ACKNOWLEDGEMENTS

The authors wish to thank Steven Porcelli for the generous gift of the CDld51.1 monoclonal antibody.

### REFERENCES

Azzoni L, Zatsepina O, Abebe B, Bennett IM, Kanakaraj P and Perussia B (1998) Differential transcriptional regulation of CD161 and a novel gene, 197/15a, by IL-2, IL-15, and IL-12 in NK and T cells. *J Immunol* **161**: 3493–3500

Bezouska K, Yuen CT, O'Brien J, Childs RA, Chai W, Lawson AM, Drbal K, Fiserova A, Pospisil M and Feizi T (1994) Oligosaccharide ligands for NKR-P1 protein activate NK cells and cytotoxicity (published erratum appears in *Nature* 1996 Apr 11 380(6574) 559). *Nature* **372**: 150–157

Burdin N, Brossay L and Kronenberg M (1999) Immunization with alpha-galactosylceramide polarizes CD1-reactive NK T cells towards Th2 cytokine synthesis. *Eur J Immunol* **29**: 2014–2025

Calabi F and Bradbury A (1991) The CD1 system. *Tissue Antigens* **37**: 1–9

Couedel C, Peyrat MA, Brossay L, Koezuka Y, Porcelli SA, Davodeau F and Bonneville M (1998) Diverse CD1d-restricted reactivity patterns of human T cells bearing 'invariant' AV24BV11 TCR. *Eur J Immunol* **28**: 4391–4397

Cui J, Shin T, Kawano T, Sato H, Kondo E, Toura I, Kaneko Y, Koseki H, Kanno M and Taniguchi M (1997) Requirement for Valpha14 NKT cells in IL-12-mediated rejection of tumours. *Science* **278**: 1623–1626

Dellabona P, Padovan E, Casorati G, Brockhaus M and Lanzavecchia A (1994) An invariant V alpha 24-J alpha Q/V beta 11 T cell receptor is expressed in all individuals by clonally expanded CD4-8-T cells. *J Exp Med* **180**: 1171–1176

*© 2001 Cancer Research Campaign* 

BJOC 01-1973 741-746 20/8/01 3:31 pm Page 746

Fujimoto T, O'Donnell MA, Szilvasi A, Yang H and Duda RB (1996) Bacillus Calmette-Guerin plus interleukin-2 and/or granulocyte/macrophage-colony-stimulating factor enhances immunocompetent cell production of interferon-gamma, which inhibits B16F10 melanoma cell growth in vitro. *Cancer Immunol Immunother* **42**: 280–284

Gillis S and Williams DE (1998) Cytokine therapy: lessons learned and future challenges (editorial). *Curr Opin Immunol* **10**: 501–503

Kawano T, Cui J, Koezuka Y, Toura I, Kaneko Y, Sato H, Kondo E, Harada M, Koseki H, Nakayama T, Tanaka Y and Taniguchi M (1998) Natural killer-like nonspecific tumour cell lysis mediated by specific ligand-activated Valpha14 NKT cells. *Proc Natl Acad Sci USA* **95**: 5690–5693

Kawano T, Cui J, Koezuka Y, Toura I, Kaneko Y, Motoki K, Ueno H, Nakagawa R, Sato H, Kondo E, Koseki H and Taniguchi M (1997) CD1d-restricted and TCR-mediated activation of valpha14 NKT cells by glycosylceramides. *Science* **278**: 1626–1629

Kawano T, Nakayama T, Kamada N, Kaneko Y, Harada M, Ogura N, Akutsu Y, Motohashi S, Iizasa T, Endo H, Fujisawa T, Shinkai H and Taniguchi M (1999) Antitumour cytotoxicity mediated by ligand-activated human V alpha24 NKT cells. *Cancer Res* **59**: 5102–5105

Kitamura H, Iwakabe K, Yahata T, Nishimura S, Ohta A, Ohmi Y, Sato M, Takeda K, Okumura K, Van Kaer L, Kawano T, Taniguchi M and Nishimura T (1999) The natural killer T (NKT) cell ligand alpha-galactosylceramide demonstrates its immunopotentiating effect by inducing interleukin (IL)-12 production by dendritic cells and IL-12 receptor expression on NKT cells. *J Exp Med* **189**: 1121–1128

Kobayashi E, Motoki K, Uchida T, Fukushima H and Koezuka Y (1995) KRN7000, a novel immunomodulator, and its antitumour activities. *Oncol Res* **7**: 529–534

Nicol AJ, Nieda M, Koezuka Y, Porcelli S, Suzuki K, Tadokoro K, Durrant S and Juji T (2000) Human invariant Vα24+ NKT cells activated by α-GalactosylCeramide (KRN7000) have cytotoxic antitumour activity through mechanisms distinct from T cells and NK cells. *Immunology* **99**: 229–234

Nieda M, Nicol A, Koezuka Y, Kikuchi A, Takahashi T, Nakamura H, Furukawa H, Yabe T, Ishikawa Y, Tadokoro K and Juji T (1999) Activation of human Valpha24NKT cells by alpha-glycosylceramide in a CD1d-restricted and Valpha24TCR-mediated manner. *Hum Immunol* **60**: 10–19

Takahashi T, Nieda M, Koezuka Y, Nicol A, Porcelli SA, Ishikawa Y, Tadokoro K, Hirai H, Juji T (2000) Analysis of human Va24+CD4+NKT cells activated by α-glycosylceramide-pulsed monocyte-derived dendritic cells. *J of Immunology* **164**: 4458–4464

Yue FY, Geertsen R, Hemm S, Burg G, Pavlovic J, Laine E and Dummer R (1999) IL-12 directly up-regulates the expression of HLA class I, HLA class II and ICAM-1 on human melanoma cells: a mechanism for its antitumour activity? *Eur J Immunol* **29**: 1762–1773

*© 2001 Cancer Research Campaign*
